# A Model of Unified Perception and Cognition

**DOI:** 10.3389/frai.2022.806403

**Published:** 2022-04-11

**Authors:** Pei Wang, Christian Hahm, Patrick Hammer

**Affiliations:** ^1^Department of Computer and Information Sciences, Temple University, Philadelphia, PA, United States; ^2^Department of Psychology, Stockholm University, Stockholm, Sweden

**Keywords:** AGI, NARS, unified model, reasoning, learning

## Abstract

This article discusses an approach to add perception functionality to a general-purpose intelligent system, NARS. Differently from other AI approaches toward perception, our design is based on the following major opinions: (1) Perception primarily depends on the perceiver, and subjective experience is only partially and gradually transformed into objective (intersubjective) descriptions of the environment; (2) Perception is basically a process initiated by the perceiver itself to achieve its goals, and passive receiving of signals only plays a supplementary role; (3) Perception is fundamentally unified with cognition, and the difference between them is mostly quantitative, not qualitative. The directly relevant aspects of NARS are described to show the implications of these opinions in system design, and they are compared with the other approaches. Based on the research results of cognitive science, it is argued that the Narsian approach better fits the need of perception in Artificial General Intelligence (AGI).

## 1. Introduction

This article refines the proposal introduced previously (Wang and Hammer, 2018), by using the ongoing design and development of NARS to introduce an approach for perception in artificial general intelligence (AGI).

AGI research pursues the goal of creating machines with human-like intelligence, though “human-like” can be interpreted at different levels of abstraction (Goertzel and Pennachin, 2007; Wang and Goertzel, 2007). For us, this means a machine which can memorize, learn, plan, reason, and pursue goals like a human does, in other words, to take intelligence as a highly abstract and general capacity (Wang, 2019).

NARS (Non-Axiomatic Reasoning System) is an AGI system designed in the framework of a reasoning (or inference) system, where the notion of “reasoning” is used in its broad sense to include many cognitive functions, including learning and perception (Wang, 1995, 2006, 2013).

Perception, usually taken to mean sensory understanding, is highly relevant to AI. Perception and cognition are inextricably linked: cognition implies information processing, and in minds new information is often sourced from the sensors, and perception is the “making sense” of sensory data in relation to existing concepts in the mind. In this way, the mind's processes may be seen as playing both perceptual and cognitive roles, the distinction mostly hinging on the abstraction and generality of the process' contents. In NARS, perception is addressed at a later stage of development, not because it is unimportant, but because we believe its design strongly depends on the existence of a general reasoning-learning mechanism, while the design and development of the latter does not depend on sensory perception to the same extent.

In this article, we introduce the ongoing work in NARS on perception and the directly related topics, with a focus on the difference of our approach with the others, especially deep neural networks. We start by clarifying our major guiding principles, and supporting them with the research results of cognitive science and the limitations of the existing AI techniques, then, the mostly relevant aspects of NARS are briefly discussed, followed by some simple examples. Finally, a few key issues in perception are discussed.

## 2. Conflicting Opinions on Perception

In Wang and Hammer (2018), we proposed three basic features on what we believe the perception process in AGI system should have: *subjective, active*, and *unified*. Here we are going to further clarify them, as well as to explain why AGI needs them and in what sense the other approaches do not have them.

### 2.1. Objective vs. Subjective

Partly because the study of perception in AI has been focused on computer vision, which grew out of the field of signal processing and pattern recognition, perception is traditionally taken as a process to describe, or model, the world or an object *as it truly is* according to the given information (Marr, 1982). The characteristics of the agent in which perception happens are widely taken as biases that should be reduced as much as possible. Such a position is also implicitly taken in deep learning, where a model aims to learn the “correct” or “best” function according to the training data (LeCun et al., 2015), though some “inductive bias” is accepted as inevitable (Mitchell, 1980).

Though this position is proper for many applications where there is well-defined correctness criteria, it conflicts with the current consensus in cognitive science, where “the existence of viewpoint-dependent representations is well established” (Jolicoeur and Humphrey, 1998). Representative opinions include:

- According to Piaget's theory, perception is the equilibration between assimilation and accommodation (Piaget, 1954), and “perception involves the mastery of the laws of sensorimotor contingencies (SMCs), those regular sensorimotor co-variations that depend on the environment, the agent's body, the agent's internal (neural) dynamics, and the task context and norms” (Di Paolo et al., 2014).
- According to Gibson's theory, our perception of an object is strongly determined by its *affordance*, that is, what we can do with it (Gibson, 1986).

In many domains, more and more researchers stress the “embodied and situated” nature of perception (Lakoff and Johnson, 1980; Brooks, 1991), which take cognition and perception as influenced by many subjective factors, including sensorimotor mechanism, conceptual framework, present situation, motivation complex, attention allocation, and so on. We believe that these factors are not only essential in human cognition, but also should be considered in perception in AGI systems.

Both in machines and in humans, perception should be taken as a selective and productive process carried out according to the current needs of the system, where incoming sensory information is organized and analyzed using the system's stored percepts (feature patterns, mental images) and concepts (notions, categories). Consequently, different systems experiencing the same situation even with the same sensors will perceive it differently, from their own subjective point of view. The same system may even experience the same situation differently if it occurs at a later time, judging the situation through a new lens according to its changed memory and attention, though similarities between the perceptions can be expected. According to this opinion, perception should not be treated as a function or computation that maps every sensory input into a unique “correct” output representation, because no “objectively correct” model of the world can be defined in the general sense; instead, each concept in the model is defined by its relations to other concepts and organized according to the system's own subjective experience.

Since the system establishes its beliefs according to its *attended* experience, new information is always filtered through the system's personal perceptual lens, including its meta-level components like the sensors, cognitive architecture, and control mechanism, which set constraints on the information actually available to and used by the system, as well as the system's current object-level belief / desire network, which itself is only a result of the system's past experience, and is subjective by definition. A perceptual agent perceiving an object is constrained in these ways when doing so, meaning an object of perception will be interpreted in different ways by different systems, and even by the same system according to different factors. So, perception is highly subjective.

This makes measuring progress in AGI perception difficult, since no one can universally define what is the “correct” way to perceive every stimulus. However, correct perception may not require a definition, but instead suffices to function as a way for the system to understand its environment and organize sensorimotor signals especially in a way that is useful relative to the (internal, subjective) goals of the perceiver. “Correct” perception is such that the system can form stable and coherent internal models of its environment and interact with it accordingly to accomplish its goals. It is not always the case that subjective knowledge is less useful than “objective” knowledge: while knowledge derived purely from individual experience may be less universal (i.e., true to all systems), more importantly it is highly relevant to the individual system and its environment. An adaptive system merely needs to learn and optimize knowledge descriptions which are useful to the system itself (i.e., in achieving its goals), not necessarily knowledge which is useful to other agents or true from a universal standpoint.

Taking the position that all knowledge is subjective does not conflict with the demand of objectivity on scientific knowledge, but rather interprets “objective” as interpersonal consensus or shared agreement between agents, as opposed to “invariably true.” By integrating various bodies of human knowledge, science can reduce the impacts of personal biases, but cannot avoid the limitations in knowledge of the whole scientific community at the current stage.

### 2.2. Passive vs. Active

In AI research, perception is normally taken to be a type of *input* process, while action as a type of *output* process, where the two processes are mostly independent of each other (Russell and Norvig, 2010; Laird et al., 2017). According to this tradition, a perception module or algorithm accepts input signals or stimuli from the outside, and processes them in certain ways (recognition, selection, generalization, abstraction, etc.), to produce some internal representation for the system to use as a representation or model of the environment (Marr, 1982; Flach, 2012; LeCun et al., 2015). Though there has been some research on “active vision” (Aloimonos et al., 1988), it is not a mainstream approach in computer vision.

On the contrary, in cognitive science the argument that “perception should be considered as action” has been raised by some researchers (Hommel et al., 2001; Kevin O'Regan and Noë, 2001; Noë, 2004; Briscoe and Grush, 2015). According to this opinion, perception and action are represented and carried out in an interwoven manner and cannot be clearly separated. In particular, the knowledge about an external action takes the form of *sensorimotor contingency*, representing the feedback or changes in perception caused by the action. The description of “active” when it comes to perception is not restricted to bodily movements, but also includes attention directing the cognitive system's internal processes: comparisons, analogies, anticipations, predictions, and any top-down management of the system's memory or sensory inputs are all active.

Externally, the perceptual system executes motor commands to move its sensory organs exploring, observing, changing, and interacting with the environment from a variety of different viewpoints, forming a feedback loop. The system actively integrates sensory feedback into its memory to construct and modify concepts, knowledge, expectations, and predictions. Without active sensors, the system only observes a “zero-dimensional” view of the world, in which the system does not explore more than one viewpoint of its environment and cannot hope to gain enough information to conceptualize reliable sensorimotor models. Through movements and exploration, the system learns to identify the relativity between objects and itself, building subjective but stable internal world models grounded by its actions, with which to compare later observations. With repeated experience to the same signals, the system can autonomously learn and construct confident internal models. Without active movement and interaction, the available information is not rich enough to easily build a meaningful understanding of the environment. Every activity provides another way in which to view the environment and new information about the relative relationships between self and perceived objects.

The internal prioritization and selection of thoughts and sensations requires attention, which may be actively directed, as the system cannot process every sensory input and combination at once if it only has a small amount of limited cognitive resources and a serial control process. In sensation alone, only basic features can be pre-processed in parallel from a stimulus, after which they are presented to a serial top-down control process. The perceiver can actively focus on specific points in their visual field to search for and recognize objects, using subsets of stimulus features along with the system's internal (active) predictions. While the entirety of a sensory stimulus is available for top-down processing, the system's attention mechanisms decide what is most important to process and what is unimportant enough to be filtered from or otherwise disregarded by the system's locus of perception (Treisman and Gelade, 1980; Wolfe et al., 2006).

We believe the same principle also applies to AI, especially to AGI. It means perception should not be taken as a process in which the system passively processes all the sensory data indiscriminately. According to this opinion, perception is not a pure input process but should instead be studied together with the activities of the system. Perception involves sensorimotor and introspective coordination where the system learns and predicts the perceptual effects of its own actions, acquiring sensorimotor contingencies. In this way, perception cannot be explicitly taught to an AI system, instead the basic framework for perceptual experience is built during a gradual and active memory organization process which occurs over its lifetime, as the system autonomously moves about learning, interacting, and gaining new experiences.

### 2.3. Modular vs. Unified

There has been a long-lasting debate in cognitive science on whether human cognition and intelligence should be considered as a unified process or a cooperation of relatively separated modules (Fodor, 1983; Prinz, 2006). Arguments for the integrated modular approach can be made including the observed apparent modularity of functionality in the human brain and the intuition that abstract concepts like mathematics and language grammar seemingly transcend the domain of sensory experiences. Modules in integrated systems also have well-defined and distinct interfaces with each other, which can make their interaction clean as well as each module's operation cycle swift and independent, allowing for parallel processing. On the other hand, there are also arguments for the unification of perception and cognition, in their representation and processing (Chalmers et al., 1992; Goldstone and Barsalou, 1998; Jarvilehto, 1998; Lakoff and Johnson, 1998; Barsalou, 1999; Shams and Shimojo, 2001).

In AI research, the mainstream opinion is to take intelligence as a collection of cognitive functions (Russell and Norvig, 2010; Poole and Mackworth, 2017) that are either loosely related in a society (Minsky, 1985) or tightly integrated in an architecture (Newell, 1990). In this framework, perception is considered as occurring at the low-level of the system near the sensory interface with the environment. The modal sensory signal is mapped to one or more abstract amodal representations (e.g., dog, cat, apple, etc.), which are sent to a cognition system that carries out more “higher-level” functions, such as reasoning. In this view, the identification of a sensation from a given modality is a process mostly distinct from the cognitive process.

In our view, perception should not be considered as carried out by a separate module that is independent of the other cognitive processes, but as closely tangled with them. In particular, many basic perceptual operations can be treated as inference, and learning in perception is not that different from learning in cognition in general. Though perception can still be considered mainly as a multi-level abstraction and generalization with a certain degree of modularity, it is not a purely bottom-up process, but heavily influenced by top-down forces, including motivation, anticipation, attention, and so on. In a system with multiple types of sensor, the integration of the modalities happens at early stages of the process, rather than until each modality-specific module completes its independent perceptual processing.

The unified approach means the system uses sensory activation inputs “as-is” in perception, conceptualizing parts of the raw sensory signal itself in the same memory as all other concepts, treating all concepts and beliefs with the same set of inference rules. A sensory stimulus should not be mapped to some representative label or amodal concept; for example, a visual image of a dog should not be used and understood within the system as a separate concept named “dog.” Otherwise, the sensations are lost and the system can only work with some abstract concept named “dog,” which has no grounding in sensory concepts. Instead, the sensory signal itself should be experienced directly by its components, which cause conceptual activations and in certain combinations enable categorical inferences, constituting the overall “currently perceived” experience. The memorized features can be used later to perform object categorization when sensing features that constitute the same or a similar pattern.

From a unified perspective, it is essential to conceptualize the sensory-perceptual items within the same memory as their productive derivations, as these lowest-level concepts are the foundation used in establishing a grounded (modal) framework for the development of further conceptual relations. Sensory processing is therefore not performed entirely by some module separate from the main system, since sensory experiences themselves need to interact directly with the system's memory. The system, of course, should be selective about what subsets of the sensory data it processes, creating abstractions and forming new beliefs that help the system competently navigate the world in service of its goals. Certain compound perceptual concepts may be constructed directly from sensory inputs and should maintain their sensory modality, meaning the perceptual concept's structure remains analogous to the structure of the sensory experience that produced it, though not necessarily isomorphic to the structure of the object in reality (e.g., a visual percept is “seeing-like” [not necessarily picture-like] in that the same concepts for visual features activate, an auditory percept is “hearing-like” in that concepts for aural features activate, etc.) as argued by Barsalou (1999).

## 3. The NARS Approach

As we have argued above, the results in cognitive science suggest that a perceptual system should be subjective, active, and unified. Non-Axiomatic Reasoning System (NARS) (Wang, 2006) basically realizes these characteristics, and so makes a natural candidate for a digital AI capable of perception. As we will see, the native logic and architecture of NARS are capable of representing perceptual concepts and allowing perceptual inferences.

### 3.1. Representation

NARS uses a *concept-centered* knowledge representation where “concept” gets a broad interpretation to represent any recognizable entity in the system's experience. Consequently, a concept may correspond to a perceived pattern, an executable operation, a word or phrase in a language used for communication, or an internal entity constructed from the above entities that are directly obtained from the system's experience. The system's memory is intuitively a network of concepts.

Each concept in NARS is named by a *term*, which is an identifier within the system to address and manipulate concepts. NARS used a term-oriented knowledge representation language, *Narsese*, which is defined in a formal grammar (Wang, 2013). The language is “formal” in the sense that a symbol in a grammar rule can be instantiated by different terms to name different concepts. However, a concept itself cannot be interpreted as different objects or events. Therefore, in NARS a concept is not a “symbol” as in the traditional symbolic AI tradition (Newell and Simon, 1976) that needs to be interpreted or “grounded” to become meaningful (Searle, 1980; Harnad, 1990). Instead, its “shape” (that is, its name) indicates its role in the system's experience, which is what it “means” to the system and decides how it will be treated by the system.

The above *experience-grounded semantics* (Wang, 2005) not only provides justifications to the logic of NARS, *Non-Axiomatic Logic (NAL)* (Wang, 2013), but also makes it possible to extend the applicable domain of this logic from abstract concepts to include concepts with sensorimotor associations, as both sensorimotor experience and linguistic experience are involved when determining the meaning of a concept or its identifier, a term.

There is a term hierarchy in Narsese to organize different types of terms, as well as the concepts they name:

- **Term**: In its simplest form, a term is merely an internal identifier of a concept, usually in the form of a string. The internal structure of such an atomic term is no longer analyzed, so can be arbitrary, but as soon as it is used to name a concept, this term-concept association becomes permanent in the system's life-cycle. In examples used in publications and demonstrations of NARS, words in a natural language, like *bird* and *crow*, are often used to give the terms (and the named concepts) some intuitive meaning, though they never exactly mean what those words mean for human speakers.
- **Variable term**: Such a term can name different concepts in different sentences, like a variable in a mathematical equation and a pronoun in a human language. The full meaning of such a term remains undetermined until it is substituted by a non-variable term, though the sentence where it is used still provides part of its meaning.
- **Compound term**: A term can be a structure formed by a few component terms plus a connector that has a predetermined meaning. For example, there are term connectors that are intuitively similar to (though not exactly defined as) the operators in set theory, such as *union, intersection, difference, Cartesian product*, etc.
- **Statement**: If a term corresponds to a conceptual relation, it is a statement that can be given a truth-value measuring the evidence in the system's experience that supports or denies the stated relation. The simplest statement in Narsese consists of two terms related by a copula and indicates that one term *can be used as the other*, in term of either meaning or truth-value. In Narsese, the basic copulas include *inheritance, similarity, implication*, and *equivalence*. There are also compound statements formed from other statements by *conjunction, disjunction, negation*, etc.
- **Event**: An event is a statement with a time-dependent truth-value, so it has a temporal attribute indicating the moment when the truth-value is about. When this attribute is specified with respect to the current moment, it is similar to the “tense” of a sentence in human languages, as it roughly indicates the occurrence time of the event, which can be *past, present*, or *future*.
- **Operation**: An executable operation of the system logically is an event that can be realized by the system itself. Operations are statements with “procedural interpretation” as in logic programming (Kowalski, 1979).

The above term hierarchy, as part of Narsese, has been fully specified in Wang (2013) to uniformly represent declarative, episodic, and procedural knowledge. Currently, the NARS team is extending this framework to fully cover the system's interaction with its environment. It means that NARS not only can communicate with other systems in Narsese, but should also use other (human or computer) languages, as well as to directly interact with its environment via sensors and actuators.

As NARS is designed to be general-purpose, it has no built-in sensor, actuator, or natural language processing module. Instead, the system can connect to various hardware or software, and use them as tools by converting certain Narsese operations as commands of that device, and returning feedback and result as input data to NARS. Consequently, perception of the environment is carried out by operations, too, as a form of active perception.

Sensations received by sensors are represented in Narsese as atomic terms, from which compound terms are constructed. Besides the term connectors mentioned previously, there are also connectors for basic temporal or spacial relations among events that construct patterns accordingly.

In summary, the concept-centered knowledge representation uniformly represents sensorimotor, linguistic, and conceptual experience, in which the system's own action plays a central role.

### 3.2. Inference Rules

NAL, the logic implemented in NARS, has been formally specified in Wang (2013), so in this article we only discuss how its usage is extended from high-level cognition to cover sensation, perception, and action.

As mentioned previously, in NAL the truth-value of a statement indicates the (extent of) agreement between the statement and the system's experience, rather than between it and the facts in the world or a model of the world. Consequently, a non-deductive inference rule (such as induction and abduction) can be justified as “truth-preserving” as long as the truth-value of its conclusion correctly measures the evidential support provided by its premises, according to the relevant definitions (of evidence, truth-value, etc.) in the experience-grounded semantics of NARS (Wang, 2005).

A simple form of temporal induction in NARS occurs when the system observes in a sensorimotor channel that event *A* is following by event *B* after a period of time *t*. When the two events are taken as premises by the **induction rule**, a conclusion “(A /⇒ B)” is derived, with *t* as a hidden attribute. This conclusion intuitively states “A is followed by B (with an time interval *t* in between).” As this conclusion is only supported by a single observation (plus that the occurrences of *A* and *B* may be uncertain themselves), the confidence of the conclusion is relatively low, so can be considered as a hypothesis.

If the succession of *A* and *B* are repeated perceived, each of them will generate such a hypothesis. With two judgments on the same statement as premises, the **revision rule** will generate a new truth-value for the statement according to the accumulated evidence, and the confidence of the conclusion will be increased accordingly. In this way, some “hypotheses” may gradually grow into “opinions,” “beliefs,” and even “facts”—in NARS, the difference among those categories are mainly quantitative (in confidence level), not qualitative (such as confirmed vs. guessed).

The inductive conclusions can be used by the other rules for various purposes. For instance, when *A* is observed again, it can be used with A /⇒ B as premises by the **deduction rule** to generate a prediction of *B* (after time *t*). If *B* indeed occurs as anticipated, A /⇒ B is further strengthened by this positive evidence, otherwise, it is weaken by this negative evidence (that is, its frequency value is decreased, though its confidence value is still increased). In this way, with the coming of more and more (positive or negative) evidence, the frequency value of the conclusion will be similar to the probability for the prediction to be confirmed, and the confidence value will become higher and higher, so the conclusion will be less sensitive to new evidence.

The above process learns a regularity from the system's experience, and is intuitively similar to statistics. However, the process is not governed by a predetermined algorithm, but formed by the cooperation of several inference rules in a data-driven and context-sensitive manner. Furthermore, the truth-value of a statement like A /⇒ B is not only determined by enumerative induction. If the system has beliefs A /⇒ C and C /⇒ B, the **deduction rule** can also derive A /⇒ B, and a deductive conclusion has a higher confidence value than an induction conclusion when the premises have the same truth-value. This is related to the discussion on “correlation vs. causation”—when a conclusion is only generated inductively, it is often considered as merely “correlative,” while a deductive justification often makes it to be accepted as “causal.” In NARS, this distinction can still be made, though it is again quantitative, not qualitative, as in NARS there is no *objective/true* cause assumed for an event.

When two terms *A* and *B* are sensory or perceptual, belief A /⇒ B can represent the system's episodic knowledge about the succession of two signals, such as sounds or images, and the above inference can happen on them, just as when they represent abstract events. When one of the two is an operation, then the knowledge represents its pre-condition (for *B*) or post-condition (for *A*), and the inference on them carries out classical conditioning (Wang and Hammer, 2015). This simple example shows aspects of the unity of cognition and perception in NARS.

According to the experience-grounded semantics of NARS, the meaning of a concept is not determined by certain sufficient and necessary condition, but by its experienced relations with other concepts. Consequently, classification and recognition are often carried out by abduction, that is, whether an instance *A* belongs to a concept *C* depends on whether it has a known property *P* of the concept. In NAL, the **abduction rule** takes C → P and A → P as premises to derive A → C. Similar to the situation of induction, in abduction each property *P* can be either positive evidence (*A* has property *P*) or negative evidence (*A* has no property *P*), and each only provides certain amount of contribution to the conclusion, rather than decides its truth-value once for all.

Beside building relations among concepts and revising their truth-values, NAL also constructs new concepts from the existing ones. For example, from A → B and A → C, a **compositional rule** can derive A → (B ∩ C), as an attempt to reorganize experience in a more efficient way. If the system experiences more such cases, the concept (*B* ∩ *C*) will be used to replace the individual usages of *B* and *C*. Initially, the meaning of the concept (*B* ∩ *C*) is completely determined by its defining relations with *B* and *C*, but gradually the compound term may form relations that cannot be fully reduced into the relations with its components.

Once again, here we can see that NAL uses the same inference rules to carry out cognition and perception.

### 3.3. Working Process

All interactions between the system and its environment happen via one of the input-output channels. Each channel connects NARS with another (human or computer) system, or a hardware/software used by NARS as a tool. A channel is invoked by certain operations of NARS, and the results of the operation are accepted into the system as input tasks.

As a reasoning system, NARS accepts the following types of *task*:

- **Judgment:** A piece of new experience to be processed. Formally, it is a statement with a truth-value indicating the evidential support of the stated conceptual substitutability. Here “statements” include sensations and perceptions.
- **Goal:** A conceptual relation to be realized by the system via the execution of some operations. Formally, it is a statement with a desire-value indicating the extent to which the system wants the statement to be true.
- **Question:** A statement with a unknown truth-value or desire-value that will be decided according to the system's knowledge.

The functions of a channel include data format conversion (such as between Narsese and RDF triples), task buffering and filtering, etc. In a sensorimotor channel, certain types of simple inference (such as temporal induction and compound event composition/recognition) are carried out on input tasks, which can be seen as the beginning of perception.

Selected tasks from the channels enter the overall experience buffer, which carries cross-modality composition to form more complicated patterns and hypotheses. Selected tasks from this buffer enter the system's memory for long-term storing and processing.

As described previously, the memory of NARS is a concept network. Beside the tasks to be processed, there is also the *knowledge* of the system, including

- **Belief**: A statement with a truth-value summarizing the system's judgments on the stated conceptual relation.
- **Desire**: A statement with a desire-value summarizing the system's goals on the stated conceptual relation.

Therefore, the knowledge of the system is its self-reorganized experience, to be used to process the tasks.

The tasks and knowledge are clustered into the concepts, according to the terms composing them. For instance, if a task or belief is about belief S → P, then it can be accessed from the concepts named by terms *S* and *P*, respectively.

NARS runs by repeating a basic working cycle. In each cycle, the channels and buffers preprocess some tasks as described previously, and in the memory a task is selected, together with some belief (or desire). The task and the belief will trigger the applicable inference rules to derive new tasks, which will be preprocessed in the internal experience buffer, then selectively enter the overall experience buffer, where they are handled together with the input tasks.

NARS opens to novel tasks in real time, which means new input tasks may arrive at any moment, and the full accomplishment of a task may require knowledge beyond the system's current knowledge scope. For example, the system may have no idea about how to realize a goal, as it demands a special sequence of operation execution that the system never did before. Under the assumption of insufficient knowledge and resources, NARS cannot simply reject such tasks, but has to try its best according to its experience (as summarized in its knowledge). However, the system cannot afford the time-space cost of exhaustively considering all relevant knowledge, because the tasks usually have response time requirements, in the form of hard or soft deadlines (such as “as soon as possible”).

The principle guiding NARS to handle this insufficiency is *relative rationality* (Wang, 2011), that is, to accomplish the tasks as much as the current knowledge and resources allow. The system dynamically allocates its processing capacity among the tasks and beliefs according to their priority values, which summarize multiple factors evaluated according to the system's experience (Wang, 2004; Wang et al., 2020).

The above overall strategy has very important implications in the perception process.

First, there is no predetermined “perception algorithm” in the system. Instead, the perception process for a given input is formed at the run time by the cooperation of multiple inference rules, as well as by the knowledge used as premises by those rules. Even repeated input may be perceived differently at different moments, given the ever changing memory structure and priority distributions among concepts, tasks, and beliefs.

The perception process in NARS is not purely bottom-up from sensory patterns to conceptual labels. Instead, sensory and perceptual terms are related to the existing concepts in the memory, including the operations. As suggested in Wang and Hammer (2018), complicated patterns will be composed with operations as components. On the other hand, knowledge about an operation is often represented by its pre-condition and post-condition, both can be in the form of sensation or perception. Consequently, how a section of experience will be perceived, that is, how the sensations are organized and classified, not only depends on the input signals themselves, but also on the available concepts (into which the sensations can be categorized) and their priority levels (that is, which have been activated by the external and internal environment). Consequently, the perception process is similar to Piaget's theory of assimilation and accommodation—what the system perceives depends on what it knows and what it is thinking about, and at the same time, its conceptual structure is more or less modified by the new experience.

Compared with other channels, a major feature of a sensorimotor channel is the huge amount of input data to be processed. It is obviously impossible to treat all sensations as concepts. Unlike the usual assumption, NARS does not attempt to “model the world as it is,” and the quality of its knowledge, including the part that is directly related to its sensorimotor interface, is evaluated according to its contribution to the adaptation of the system, especially its relationship with the tasks. Only “high quality” perceptive patterns are entered into memory as concepts, and the “quality” of a perceptive pattern depends on the following factors:

- Its **occurring frequency**: a recurring pattern deserves to be considered as a unit, as using it can describe the experience in a more compressed manner.
- Its **contribution** to the tasks: a pattern that has been useful in task processing is more likely to be useful again in a relatively stable environment.
- Its **simplicity**: under time pressure, a simple description of the situation is often preferred, even when it is less accurate than the complex alternatives.

The above factors are obviously related to the Gestalt Principles summarized from human perception (Koffka, 2014). The evaluation of these factors cannot be done once for all, but is an important part of the system's lifelong learning process.

### 3.4. Simple Examples

We want to experimentally test our perception hypotheses in NARS to evaluate their potential validity, following in technical detail the inference processes from low-level sensory data through to object categorization or classification. Since NARS is strongly influenced by the results of cognitive science, it should naturally implement perceptual processes as long as sensory stimuli (the inputs) are expressed in a readable and useful format. To demonstrate its basic perception functionality, we tested NARS' hand at an existing computer vision benchmark for classification, the MNIST database (containing labeled 28-by-28 pixel images of handwritten digits 0–9).

**Figure 1:**
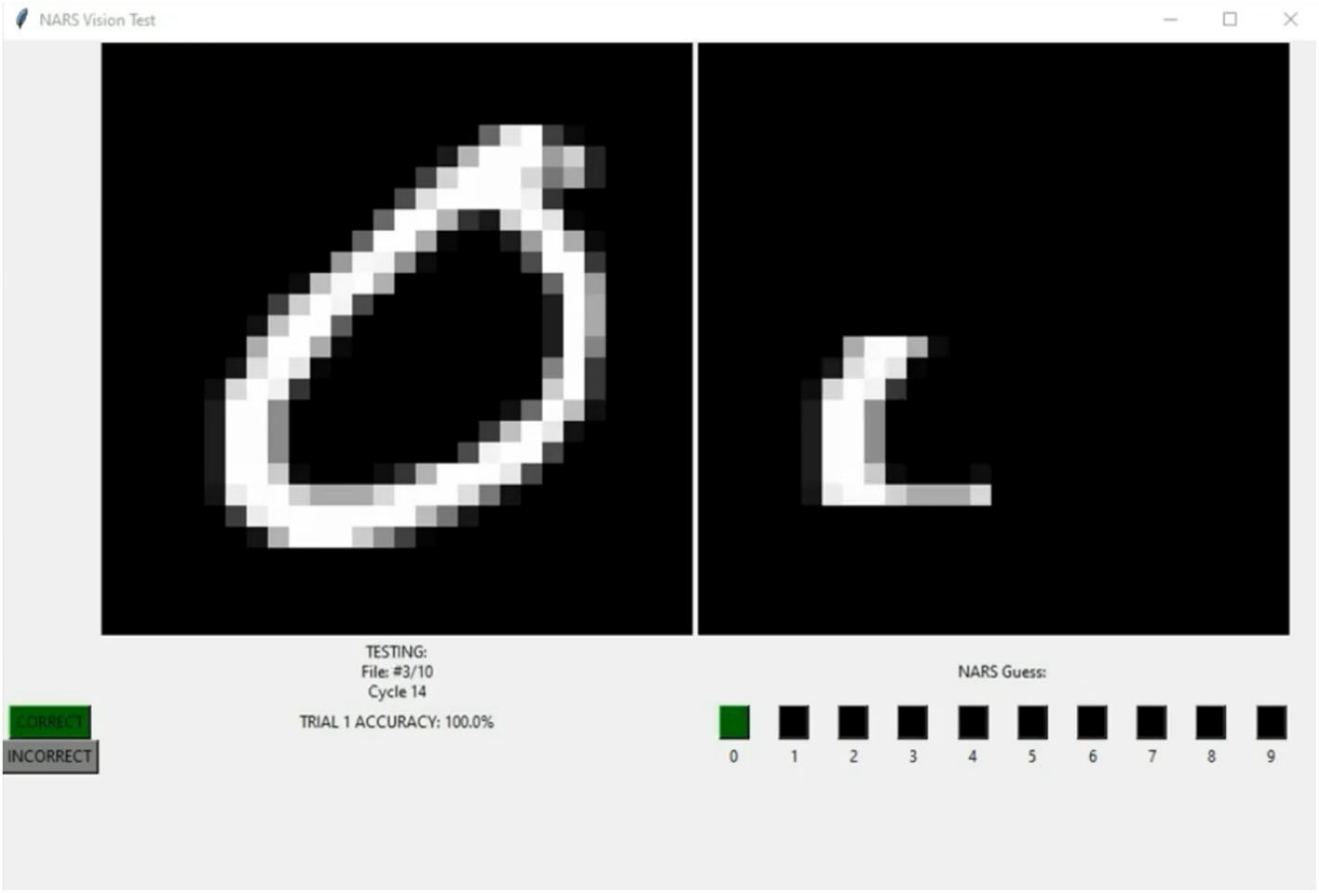
In this screenshot, NARS executes an operation indicating it recognizes an image of digit zero. The videos documenting the trials in the table are available on YouTube (Binary Memorization; Digit Memorization; Binary Classification; Digit Classification).

Here we supply preliminary results which may serve as a baseline to evaluate NARS on perception problems and compare NARS' accuracy to other AI models in this domain like convolutional neural networks (ConvNets), though we should expect ConvNets to outperform NARS in this area since they are specifically designed for image classification tasks. NARS has the advantage of being able to use its visual knowledge generically, across modalities and domains, though cannot be evaluated in exactly the same way as a neural network, since NARS is not guaranteed to produce an output for every given input. Therefore, we use the closest equivalent in NARS, executing an *operation*, which is when the system produces an output signal to perform an action (like pressing a certain button when it recognizes the shown digit). This adds complexity to the endeavor of evaluating NARS, but also allows us to evaluate the system's goal-handling abilities and autonomy. The system must of its own “volition” execute an action indicating that it recognizes a digit presented in the visual image. Therefore, the system could feasibly fail to recognize a digit at all, executing no actions, though it might try to make a reasonable guess according to the parts of the image it does recognize, as in Figure 1.

#### 3.4.1. Spatial Buffer and Feature Subset Extraction

An existing open-source NARS implementation *NARS-Python v0.3* (Hahm, 2021) was modified to test the perceptual framework developed above. Specifically, a spatial (multi-dimensional) buffer was implemented based on the previous work of array or sensory terms (Wang and Hammer, 2018) as a way to retain the topographical mapping of input events (i.e., the spatial structure of individual feature activations). The buffer holds a 2D array which is the feature map, where each element is an event representing the feature activation strength at each location.^1^ Each atomic feature is encoded as an event with a truth value, aka an *atomic sensation*. Each sensation's subject term is named according to its location, so that there is a unique term assigned to each portion of visual space. The *frequency* of the truth-value represents the sensation's strength and the *confidence* represents something like the reliability of the stimulus transduction. Events with frequency below 0.5 are negated to make their frequency above 0.5, converting their negative evidence to positive evidence for the system to better utilize. The system's modality-specific sensors are responsible for producing these atomic sensations from external stimuli and presenting them in a topographical mapping, that is a spatially ordered manner. A small subset of feature activations is extracted from the buffer and composed as a conjunction of events.

The question of prioritizing feature subset selection is one of attention allocation: (1) which features to attend, and (2) how many features? In vision, the spotlight model of attention is a theory of prioritizing feature selection where an attentional spotlight, thought to be circular/oblong or square/rectangular, jumps to especially salient features in the visual field and sharply prioritizes the processing of features within the spotlight's bounds. This exact nature of this spotlight, how it moves, what is its size / shape and how do those change, etc. is an active research question in psychology (Cave and Bichot, 1999). We can focus anywhere on the image like a spotlight by applying a 2D priority mask with a configurable sharpness or *focus* ≥ 0 in a function that elevates the relative priority of elements in the focused area by suppressing the confidence / priority of elements in the peripheral area. Specifically, the confidence of a visual feature at *relative* pixel coordinate (*x, y*) ∈ [−1.0, 1.0] × [−1.0, 1.0] within a spatial buffer from origin at (0, 0) may be defined by multiplying a unit amount of evidence with a parameterized 2D Gaussian function $c\left(x,y\right)=\frac{1}{1+k}*e^{-\left(focus^{2}\right)\left(x^{2}+y^{2}\right)}$, or priority can be defined with the Gaussian alone.

In each working cycle, the system uses attention mechanisms to extract a “patch” subset of spatially-near features from the vision buffer, also in a spotlight-type manner, though in practice any subset of features may be selected so long as their combination tends to be recognized by the system. A high-priority feature is selected probabilistically using *bag* (Wang, 1995), ranked according to its frequency and confidence (where *priority* *p* = *f* * *c*, so that very highly activated and confident features, with values closer to one, are preferred for selection), and a box of a configurable radius provides bounds for the spatial subset selection. The selected subset of events are then combined into a single compound term, a conjunction^2^; the selected feature subset can be potentially very large, (in our case, maximally 28 * 28 = 784 events) or very small (2 * 2 = 4 events) depending on the constraints of feature selection, but afterwards they may be combined and treated as a singular whole. At this point perhaps is where the lowest level of abstraction and classification occurs in the system. Multiple events are represented using a single compound term; this term names a specific concept in memory. Therefore, an abstraction occurs as the system extracts a subset and combines multiple smaller components into a single representative term of some granularity; the compound is not an arbitrary mapping, since it is directly composed of a subset of modal events. This process in NARS also helps to resist noise in the input, since fuzzy truth-value expectations for feature detections are “rounded” to their purely positive or negative form. A primitive classification occurs immediately as well, when the constructed compound is processed by NARS and activates its corresponding concept in memory. If the concept does not yet exist, a new one must be created in the memory; although there are an extremely large number of *possible* boolean feature map activation patterns (for just the singular whole 28 × 28 image, 2^28*28^ = 2^784^ = 1.02 * 10^236^), NARS only stores the smaller positive feature subsets to which it actually attends, and deletes old concepts which are rarely encountered or used (such as only once or twice in the system's lifetime) in the case that the system's memory is at capacity, ensuring the system's memory size and resource usage remains bounded at all times.

After the NARS system was programmed with spatial buffer and attention capabilities, the next step of supervised learning involved teaching class labels for each training image. During training, a label event (*L*) was input every few working cycles while at the same time the corresponding image of the numeric digit was shown to NARS. We can follow the inference process that occurs when NARS encounters an image of Digit 1 in the Training and Testing Phases.

During Training: *For compactness, we will write each first-order feature event using a single letter and index subscript, like* *E*_*y,x*_ = < {*VisionSensor*_*y*_*x*} → [*bright*]>

*// NARS is drawn to select a salient feature near the top-center of the visual field (location (7,15)), and extracts a 3x3 patch around it*.


$$\left[\begin{array}{ccc} \neg E_{6,14} & E_{6,15} & \neg E_{6,16}\\ \neg E_{7,14} & E_{7,15} & \neg E_{7,16}\\ \neg E_{8,14} & E_{8,15} & \neg E_{8,16} \end{array}\right]$$

*// The positive feature activations are conjunctively combined using a conditional composition inference rule:*


$$V_{0}=<E_{6,15}\wedge E_{7,15}\wedge E_{8,15}>.\;\langle 1.0,0.9\rangle$$

*// NARS observes our label event for the current image (digit 1)*


$$L_{0}=<digit1\to \left[seen\right]>.\;\langle 1.0,0.9\rangle$$

NARS creates temporal associations between its visual extractions (*V*_0_) and the label event (*L*_0_) when they both enter the system's temporal module. As the system further analyzes the visual image, more of these associative connections form.

*// By temporal **induction**, NARS associates the two into a predictive implication*


$$\begin{array}{l} \left\{V_{0},L_{0}\right\}\;⊢\;P\\ =<<E_{6,15}\wedge E_{7,15}\wedge E_{8,15}>/\Rightarrow <digit1\to \left[seen\right]>>.\;\langle 1.0,0.45\rangle \end{array}$$

During Testing, when the system extracts a familiar feature compound and with which it has made a temporal association with our label event, the system can infer the label from the image.

*// The same subset of visual features is selected within a slightly shifted and scaled (5 rows x 3 columns) attention window, say from a different image of digit 1 (at location (6,15))*.


$$\left[\begin{array}{ccc} \neg E_{4,14} & \neg E_{4,15} & \neg E_{4,16}\\ \neg E_{5,14} & \neg E_{5,15} & \neg E_{5,16}\\ \neg E_{6,14} & E_{6,15} & \neg E_{6,16}\\ \neg E_{7,14} & E_{7,15} & \neg E_{7,16}\\ \neg E_{8,14} & E_{8,15} & \neg E_{8,16} \end{array}\right]$$

*// The positive features are combined using conjunction, resulting in the same compound as previously encountered*.


$$V_{1}=<E_{6,15}\wedge E_{7,15}\wedge E_{8,15}>.\;\langle 1.0,0.9\rangle$$

*// After matching it to the concept, system finds a related premise in memory, recovering the predictive implication created earlier, and performing conditional **deduction** to derive the associated label*.


$$\left\{V_{1},P\right\}\;⊢\;L_{1}=<digit1\to \left[seen\right]>\;\langle 1.0,0.405\rangle$$

*// Seed goals for NARS; NARS wants to press a digit button when its corresponding digit is seen*.


$$\begin{array}{l} G_{0}=<<digit0\to \left[seen\right]>,⇑pressDigit0>!\;\langle 1.0,0.9\rangle \\ G_{1}=<<digit1\to \left[seen\right]>,⇑pressDigit1>!\;\langle 1.0,0.9\rangle \end{array}$$

*// When the label event is true, NARS can use it in **deduction** with a seed goal*.


$$\left\{L_{1},G_{1}\right\}\;⊢\;G^{\prime }=\;⇑pressDigit1!\;\langle 1.0,0.267\rangle$$

Finally, the derived *operation* goal has some *expected desirability* (in our example *d* = 0.63 though in reality it depends on the amount of evidence) which will accumulate with other desires by the Revision Rule. Eventually, if enough evidence is derived from these between the stimulus and the predictive beliefs (“perceptual committees”), and the seed goals, a very strong desire for the operation will be derived which exceeds NARS' Decision-Making Threshold, causing NARS to execute the operation.

#### 3.4.2. Experiment Methodology and Results

We evaluated NARS on four visual recognition tests. Firstly, binary *memorization*: testing the system's ability to memorize images of bits, and recall the same images in the near future. Second, digit *memorization*: the same, but with ten different digits (0 through 9). Thirdly, binary *classification*: testing the system's ability to identify *new* instances of the same class using only the knowledge it learned from labeled training examples it has seen. Finally, digit *classification*: the same as in binary, but with all ten different digits.

The overall results for our NARS visual recognition experiments are recorded in Table 1. Since NARS follows a different paradigm than neural networks, a separate (but similar) experimental methodology was developed. The experiment is divided into the familiar training and testing phase with unique random images from the dataset. NARS is exposed to each training image for a certain number of working cycles (recorded in the table), while the training example's label event is simultaneously provided as input. The training phase is followed by the testing phase, where each test image is presented to NARS (after a short priming period) until the system is confident enough to make a guess. If NARS makes no guess by a certain timeout period (here we used 3,000 working cycles), the example is counted as incorrect.

**Table 1:** Accuracy results recorded for the NARS MNIST digit memorization and classification tests (3 trials).

**Test name**	**Number of train/test images**	**Training cycles (per image)**	**Trial 1**	**Trial 2**	**Trial 3**	**Overall avg. accuracy**
Binary memorization [0, 1]	10, 5 per digit	150	100%	100%	100%	100%
Digit memorization [0−9]	10, 1 per digit	1,500	100%	100%	100%	100%
Binary classification [0, 1]	30/90	750	97.78%	98.89%	96.67%	97.78%
Digit classification [0−9]	300/100	125	48.0%	43.0%	40.0%	43.66%

There are many factors that impact the evaluation of NARS, including the system's specific design, and the system's configurable “personality parameters” which the user sets for each individual instance of NARS, and just as no two humans with different personalities would perform the same way on these tests, neither will two NARS. Variable “personality” impacts the control process and so the results of the evaluation, but this can be used to the NARS user's advantage since the user can identify more or less optimal personality parameters for the task at hand. Specifically for our digit recognition tests, we accounted for *cautiousness* (*T*), *evidential horizon* (*k*), *event* and *desire* confidence decay rates, as well as a visual *focus* which determines the confidence / priority mask on the visual field, though in future versions this value should dynamically change according to sensory saliency and NARS' motivation. In the case of the NARS program, the perceptual principles were implemented as described in this paper, with some restrictive modifications made to the system to prevent extra inference that was not strictly necessary to the test. We optimized configurable parameters (see Table 2) for each test using a greedy walk function. The accuracy values in the table are some initial results we achieved within our time frame for this research; they may serve as a baseline score to beat for future versions of NARS.

**Table 2:** Configurable NARS parameters (up to 3 decimal places) used during each test.

**Test name**	**Evidential horizon (k)**	**Cautiousness (T)**	**Priority mask focus**	**Event time decay**	**Desire time decay**
Binary memorization [0, 1]	7	0.600	20.0	0.070	0.999
Digit memorization [0−9]	22	0.582	0.159	0.560	0.999
Binary Classification [0, 1]	22	0.582	6.380	0.913	0.832
Digit classification [0−9]	1	0.65	0.935	0.95	0.95

NARS was able to achieve high accuracies on the first three tests with the simple approach alone, however the “pixel-perfect” approach was not performing as well with the more complicated digit classification task, perhaps because although feature subsets extracted from the training examples might also be present in the test examples, the exact activation pattern might be translated slightly (even just a single pixel), or there might be variations in local regions of the image (in terms of which specific features are activated), which means NARS would categorize it as a new pattern. This is a known problem in computer vision which can be remedied by “max pooling,” a procedure performed on the image features to downsample them into a more compact and robust representation (at the cost of specificity and information) which introduces a small local translation invariance in the feature representation. In max pooling, small (2 × 2) patches of the image are selected and summarized with one value, that is the maximum value from the patch. We can achieve a similar max pooling effect in Non-Axiomatic Logic using *disjunction* between features: if any of the features in some decided pool have *positive* frequencies (above 0.5), the frequency of their disjunction will be positive, so create a spatial *disjunction* of all the statements. The disjunctive “pool” is positive if *any* of its elements are positive, so is the same value regardless of any feature activations shifting within its bounds. If all of the features in the pool have *negative* frequencies (below 0.5), then the frequency of their disjunction will be negative, so they may be represented as a negated *disjunction* of positive terms, or equivalently a *conjunction* of negated terms.

*Say we have a 4 x 4 feature map:*


$$\left[\begin{array}{cccc} \neg E_{13,14} & E_{13,15} & \neg E_{13,16} & \neg E_{13,17}\\ \neg E_{14,14} & E_{14,15} & \neg E_{14,16} & \neg E_{14,17}\\ \neg E_{15,14} & E_{15,15} & \neg E_{15,16} & \neg E_{15,17}\\ \neg E_{16,14} & E_{16,15} & \neg E_{16,16} & \neg E_{16,17} \end{array}\right]$$

*Nearby features can be combined disjunctively as a form of max pooling (in this case, 2 x 2 pooling with stride 2, resulting in a 2 x 2 pooled feature map from which elements can also be selected):*


$$\left[\begin{array}{cc} <E_{13,14}\vee E_{13,15}\vee E_{14,14}\vee E_{14,15}> & \neg <E_{13,16}\vee E_{13,17}\vee E_{14,16}\vee E_{14,17}>\\ <E_{15,14}\vee E_{15,15}\vee E_{16,14}\vee E_{16,15}> & \neg <E_{15,16}\vee E_{15,17}\vee E_{16,16}\vee E_{16,17}> \end{array}\right]$$

## 4. Discussions of Key Issues

In the following, we compare the proposed perception mechanism of NARS with the other approaches on how a few key issues are handled.

### 4.1. Multi-Level Abstractions

Perception is widely regarded as a multi-level abstraction or generalization, which gradually turns sensory signals or stimuli into concepts and their relations. Though this opinion is shared by almost every approach in artificial intelligence and cognitive science, there have been many very different ways to carry out the process (Marr, 1982; Zhu and Mumford, 2006; Stone et al., 2017).

On this topic, the most successful model so far is Deep Neural Networks (DNN) (LeCun et al., 2015). For the current discussion, this model, in its typical form, can be characterized as the following:

- The model is constructed as a network consisting of multiple layers of processing units, or *neurons*. There are predetermined links among the neurons, though the strength or *weight* of each link is learned from training data.
- Each neuron is a parameterized function that carries out of a certain mapping from input to output, which can be seen as an abstraction or generalization, since the mapping is usually many-to-one. In the whole model, there is only a small number of neuron types, though for each type, there are typically a large number of neurons.
- The learning process follows a predetermined algorithm, which adjusts the parameters (mainly the weights) gradually to approximate or optimize a target function that is exemplified by the training data.
- After learning (training), the model is used as a fixed mapping from an input layer to an output layer, with multiple hidden layers in between, those meaning usually cannot be explained independent of their contribution to the overall mapping.

While many models before DNN depend on the human designers to pick the features to be abstracted in each layer, DNN learns them by considering the contribution of each candidate to the overall function. Thanks to the universal approximation power of this model, the huge among of training data, and the great computational power of new hardware, this approach has achieved great successes.

Compared to DNN, the approach we are promoting shares the idea of feature learning, though it is carried out in a very different way:

- The knowledge of NARS is organized in a network of interconnected processing units, or *concepts*. Both the topological structure and the network parameters are learned from experience.
- A concept summarizes an ingredient or pattern in the system's experience in terms of its relations with other concepts, which is what it *means* to the system. This meaning is obtained and grounded in the system's experience, which serves in the system's treatment to various task. A concept is neither a function (mapping an input vector to an output vector) nor a symbol (denoting an external object).
- In NARS, each concept provides a type of abstraction by relating some other concepts in a specific way. This relation can be temporal, spatial, compositional, or substitutable (in meaning or truth-value). These relations are closer to the relations among human concepts than the nonlinear version of the weighted sum used in DNN. This makes NARS more explainable, including when the system's conclusion is wrong, unlike when a DNN meets its adversarial examples.
- In NARS the reasoning-learning process is data-driven and selective, whereby only relevant conceptual relations are updated by carrying out inference and revision. This feature makes the system open to novel tasks, though no satisfied solution is guaranteed to all of them. In NARS, the perception process is usually not exactly repeated even for same input. Though a certain level of repetition, especially for bottom-up processes, might be achieved.
- In NARS, there is no distinction between training and operating phases, as learning continues with every new example presented, though of course it is possible to test the system on new examples if desired. Consequently, NARS is not restricted to stationary environments specified by the training data, and new examples are not demanded or assumed to come from the same sample space or population as in statistics.
- For DNN's, overfitting can occur when the model is trained for too long on examples of the training data and does not contain information bottlenecks which make it unable to remember a large number of specific inputs. Since NARS is not forced to update a single model but can maintain competing hypotheses with different evidential bases, it is less prone to overfitting, though it can still happen when there is no space left for more general hypotheses or not enough resource to build them in the first place. Additionally, limits in memory sizes act as a natural information bottleneck which make it usually impossible for the system to remember every input.

A primary aspect of perception is identifying “high-level” categories and concepts from a given “low-level” stimulus using abstraction and generalization. By abstraction, we mean reducing complexity using a single entity to represent a combination of multiple smaller parts (e.g., viewing “the bigger picture” and ignoring the minute details), and by generalization, we mean identifying more general categories from more specific categories. These processes go hand-in-hand, and are naturally supported by NAL inference, which defines “category” or “inheritance” as the basic relationship between concepts and allows compounding. What happens in the system during perceptual abstraction? If there are multiple items from a sensory buffer or memory which are combined and treated singularly such as in conjunction, an abstraction has occurred. In NARS memory, each concept is related to some others according to experience in a graded generalization hierarchy, where concepts of all different abstraction and generalization levels exist and may be activated during perception. With this in mind, we can attempt to weakly delineate what is a “high-level” or “low-level” concept, and how the two are bridged together with “mid-level” perception (composition and categorical inferences).

There are no explicitly defined generalization or abstraction levels in the NARS conceptual hierarchy except the extreme terminals: *properties* which describe the most general category and *instances* which describe most specific. Instead, concepts are networked in such a way to form a *gradient* of generalization, and multiple concepts can be represented within a single more abstract concept. There is no hard distinction between high-level and low-level concepts, but we can attempt to classify them based on their historical origin and structure: (1) the perceptual process begins with sensor detections at the point of interaction with the environment, from which the system extracts organism-specific “hardcoded” features from the sensory stimulus in parallel, which can activate their corresponding **low-level** atomic concepts, (2) a spatially-ordered map of these “pre-processed” activations is presented or otherwise made available to the system's top-down control process, which uses factors like attention to select features and compose them as a compound which may match with a corresponding **mid-level** concept in memory, (3) finally, when many low- and mid-level concepts have been created in the memory and associated via reasoning, the system can further combine and generalize those underlying concepts to construct “perceptually grounded” **high-level** abstract concepts.

In summary, while sharing certain ideas with the other approaches, in NARS the multi-level abstraction process in perception is carried out in a very different way. While NARS also accepts raw sensory data and generalizes / abstracts it like the neural network, units of all specificities are treated conceptually within the system from the lowest to the highest levels of generalization and abstraction. Instead of combining all low-level information at once with an algorithm of different synaptic weights to classify a more abstract object, the system continuously, incrementally, and selectively extracts subsets of sensations which it compares to its memories, helping to identify contextually relevant actions and knowledge.

### 4.2. Perception as Inference

To consider perception as inference is not a new idea, though the previous works mostly use this idea to explain human perception (Rock, 1983; Hatfield, 2002; Hockema, 2004; Shanahan, 2005).

What we have been doing is to give this idea a constructive proof by accomplishing perception in a reasoning system implemented in a computer. Roughly speaking, in NARS

- disambiguation and contradiction resolving are typically done by the revision rule and the choice rule,
- recognition and explanation are typically done by the abduction rule and the revision rule,
- generalization and abstraction are typically done by the induction rule and the revision rule,
- prediction and demonstration are typically done by the deduction rule,
- concept creation is typically done by the term composition rule.

In our view, the bridge between low-level signals and high-level categories can be achieved by inference. Perception fits naturally within a framework of inference processes, since both seek to construct meaningful results from components or premises. For example, a primary “perceptual inference” is the process of categorization, where the system uses its many concepts to guess a more abstract category of a subset of its sensations. What the system perceives during its operation is the experience of many combined inference results; these “perceptual committees” (Wolfe et al., 2006) or internal schemata inform the system's perceptual beliefs, each chipping in their preferred logical suggestions on how to interpret a stimulus. Inference results and predictions will tend to complement when they agree, say allowing the system to recognize an object partially occluded by a tree. When inference results conflict, the more confident results will tend to dominate the others, which becomes apparent in the case of certain illusions where our minds are “tricked” by our senses.

In order to help with constructing useful mid- or high-level concepts or schemata as well as make categorization simpler, the system has built-in bottom-up processes that automatically extract useful low-level perceptual features in parallel from an incoming stimulus. Which features are extracted impose a constraint on what is possible for the system to perceive. Extracted “atomic” features will activate their corresponding concepts during the “pre-processing” of a sensation, which helps guide the system's top-down selections and comparisons to other parts of memory. In this way, perception is very context-dependent, its content relying heavily on both the signals from the current environment as well as the system's attention and current memory structure. The reasoning process produces compounds and/or predictive statements which constitute the system's mid-level and high-level concepts. Later, when the system experiences many of the same low-level features again, it can use the activated features together with the previously learned associations to categorize the stimulus. Various factors decide the exact concept and stimulus selections during perception, which constitute the system's attentional mechanisms. The system can then use other top-down mechanisms such as prediction and anticipation to confirm or disprove its categorical beliefs. The system over time compiles evidence for and against relationships between low-level features and higher-level concepts, ideally optimizing the system's knowledge in terms of helping it achieve its goals.

Compared to the common algorithm-guided processes, rule-based processes are more naturally justifiable and support real-time responses (Wang and Li, 2016; Wang et al., 2020), though NARS can also use problem-specific algorithms as tools or “organs” (Hammer et al., 2021).

### 4.3. Realization of the Principles

The above description about NARS provides a concrete framework for the principles of perception discussed at the beginning of the article.

The perception process in NARS is ***subjective***, mainly because it is based on experience-grounded semantics. According to this theory, the meaning of a concept is determined by what the system knows about it, and the truth-value of a statement is determined by how much (positive and negative) evidence the system has about it. The above treatment is fully applied to perception, where the concepts and beliefs are from the system's point of view. In particular, the system's perception produces beliefs similar to the sensorimotor contingency studied in cognitive science, which describes the world or environment in the form of “If I do *A*, I am going to see *B*,” which is completely from the system's perspective. Even so, we can also explain how objectivity gradually comes into the system's knowledge. As soon as NARS starts to interact and communicate with other cognitive systems, it may observe similar relations among events and other's actions. As a result, some egocentric beliefs will be generalized, such as from “If I do *A*, I am going to see *B*” to “If X does *A*, X is going to see *B*,” where X can be any agent. Such a general belief can be shared via communication and be strengthened by the positive feedback from the others, and gradually become part of the common knowledge, or “commonsense” of a society. Some of the knowledge may even be projected to the world, like “*A* causes *B*,” as if it does not depend on any agent or cognitive system. In this way, perception in NARS is primarily and fundamentally subjective, though can gradually include objective factors in it.

The perception process in NARS is ***active***, since knowledge is established relative to the system's actions, and perceptual information is selected attentively. Top-down operations are normally invoked by the system to accomplish existing tasks, though some can also be automatically triggered by strong external stimuli. In NARS, sensors and actuators are handled similarly, as both provide feedback to the system as input information, though we can still say that the main job for a sensor is to collect information, while for an actuator is to make a change. Our future plan for vision is not analyzing static images, but to use sensors to actively “scan” and navigate a whole environment. Naturally, complicated perceptive patterns will be represented as scan trace (Wang and Hammer, 2018), or compound terms with both sensory and motor components. Active perception occurring within the system's internal working space means the system might be able to obtain self-awareness and self-control via introspection, which raises the possibility of consciousness, as analyzed in Wang (2020).

The perception process in NARS is ***unified***, simply because the same memory, control, and set of inference rules are used for perception and cognition. Of course, perception still needs special treatment, which is what we are still on, for several reasons. First, the raw data coming into the system has different formats. For example, vision typically gets input data from a large number of light sensors, though the exact features of these sensors may be different in different systems (both human and computer), and likewise for other modalities. We need to find efficient ways to represent features and to preprocess them, so as to turn them into terms similar to those from other channels. As far as inference is concerned, the challenge here is that the inputs from a sensorimotor channel are mainly related by spatial and temporal relations, rather than by compositional (syntactic) and semantic relations, as knowledge from linguistic channels. Even though the same set of inference rules are used, special attention allocation policy is needed to efficiently select the useful patterns and contingencies among the huge number of candidates.

One important consequence of this unification of perception and cognition is that the widely accepted distinction between “symbolic” and “subsymbolic” disappears in NARS. As explained previously, the concepts of NARS are not symbols representing external objects and events, but directly grounded on the system's experience, they share many properties with the processing units in connectionist networks (Smolensky, 1988), though they are not like neurons. There are still concepts in NARS that are directly associated with sensors and actuators, as well as concepts that are only remotely related to sensorimotor, but their difference is quantitative and relative, not qualitative and absolute, as widely assumed. NARS does not integrate these two paradigms into a hybrid “neuro-symbolic” model, though share some ideas with both at different places.

## 5. Conclusions

Perception in NARS is still an ongoing endeavor with many open problems. Even so, our progress so far shows the potential of applying certain well-established principles reached in cognitive science, namely taking perception as subjective, active, and unified with cognition.

On the technical level, our work shows the potential of carrying out perception as reasoning, especially in NARS, a model built on the foundation that intelligence and cognition can be taken as *adaptation with insufficient knowledge and resources*.

We believe our lessons can contribute to better understand of perception, as well as to general intelligence.

## Data Availability Statement

The original contributions presented in the study are included in the article/supplementary material, further inquiries can be directed to the corresponding author.

## Author Contributions

PW proposed the structure of the article and the major contents. PH contributed to the inference examples. CH added discussions about human perception and carried out the experimental trials. All authors contributed to the description of the NARS approach of perception.

## Funding

The research is partly funded by a gift from Cisco Systems, Inc. to support our research in Cognitive Automation via Reasoning and Learning. The funder was not involved in the study design, collection, analysis, interpretation of data, the writing of this article or the decision to submit it for publication.

## Conflict of Interest

The authors declare that the research was conducted in the absence of any commercial or financial relationships that could be construed as a potential conflict of interest.

## Publisher's Note

All claims expressed in this article are solely those of the authors and do not necessarily represent those of their affiliated organizations, or those of the publisher, the editors and the reviewers. Any product that may be evaluated in this article, or claim that may be made by its manufacturer, is not guaranteed or endorsed by the publisher.

## Footnotes

^1^In the future, the buffer can be fitted with a 3rd dimension to hold other feature maps like color, edge orientation, etc.

^2^*F*_*int*_ (Wang, 2013) was modified to use a version of extended Boolean function $and\left(x_{1},...,x_{n}\right)={\left(x_{1}*...*x_{n}\right)}^{\frac{1}{n}}$ which works like geometric average, maintaining higher confidence for conjunctions of even large quantities of events.
